# Physiological and Biochemical Response to *Fusarium culmorum* Infection in Three Durum Wheat Genotypes at Seedling and Full Anthesis Stage

**DOI:** 10.3390/ijms22147433

**Published:** 2021-07-11

**Authors:** Jakub Pastuszak, Anna Szczerba, Michał Dziurka, Marta Hornyák, Przemysław Kopeć, Marek Szklarczyk, Agnieszka Płażek

**Affiliations:** 1 Department of Plant Breeding, Physiology and Seed Science, University of Agriculture, Podłużna 3, 30-239 Kraków, Poland; anna.szczerba21@gmail.com (A.S.); marta.hornyak@botany.pl (M.H.); rrplazek@cyf-kr.edu.pl (A.P.); 2 Franciszek Górski Institute of Plant Physiology, Polish Academy of Sciences, Niezapominajek 21, 30-239 Kraków, Poland; m.dziurka@ifr-pan.edu.pl (M.D.); p.kopec@ifr-pan.edu.pl (P.K.); 3 Polish Academy of Sciences, W. Szafer Institute of Botany, Lubicz 46, 31-512 Kraków, Poland; 4 Faculty of Biotechnology and Horticulture, University of Agriculture, 29 Listopada 54, 31-425 Kraków, Poland; marek.szklarczyk@urk.edu.pl

**Keywords:** antioxidant enzymes, deoxynivalenol, *Fusarium culmorum*, mycotoxins, nivalenol, *Triticum durum*

## Abstract

*Fusarium culmorum* is a worldwide, soil-borne plant pathogen. It causes diseases of cereals, reduces their yield, and fills the grain with toxins. The main direction of modern breeding is to select wheat genotypes the most resistant to *Fusarium* diseases. This study uses seedlings and plants at the anthesis stage to analyze total soluble carbohydrates, total and cell-wall bound phenolics, chlorophyll content, antioxidant activity, hydrogen peroxide content, mycotoxin accumulation, visual symptoms of the disease, and Fusarium head blight index (FHBi). These results determine the resistance of three durum wheat accessions. We identify physiological or biochemical markers of durum wheat resistance to *F. culmorum*. Our results confirm correlations between FHBi and mycotoxin accumulation in the grain, which results in grain yield decrease. The degree of spike infection (FHBi) may indicate accumulation mainly of deoxynivalenol and nivalenol in the grain. High catalase activity in the infected leaves could be considered a biochemical marker of durum sensitivity to this fungus. These findings allowed us to formulate a strategy for rapid evaluation of the disease severity and the selection of plants with higher level, or resistance to *F. culmorum* infection.

## 1. Introduction

**Figure 1:**
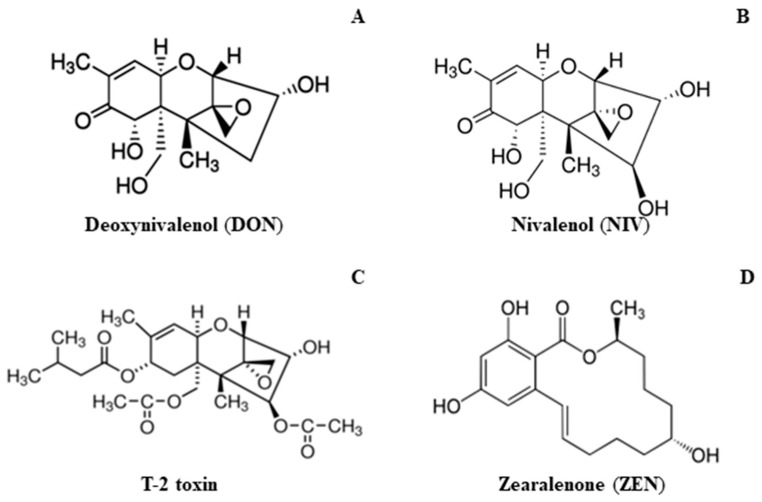
Secondary metabolites (mycotoxins) produced by *Fusarium culmorum*: (**A**) deoxynivalenol (DON), (**B**) nivalenol (NIV), (**C**) T-2 toxin, (**D**) zearalenone (ZEN).Source: Sigma-Aldrich.

**Figure 2:**
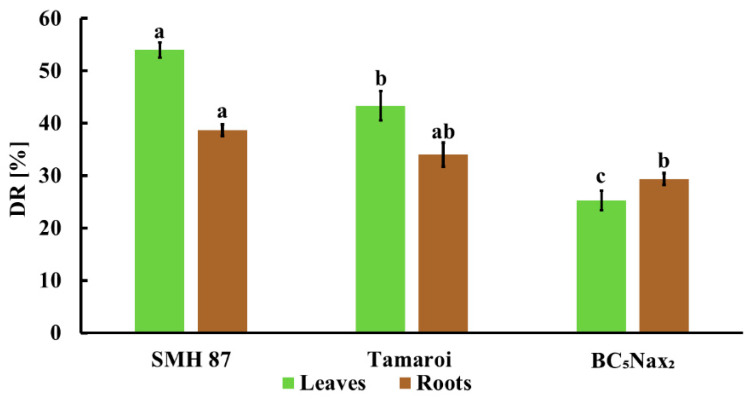
Disease rating (DR) in the leaves and roots of three durum wheat genotypes infected with *F. culmorum*. The values represent means (*n* = 30) ± SE (standard error). Different superscript letters (a–c) for each organ indicate significant differences between means (Duncan’s multiple range test; *p* < 0.05).

Fungi of *Fusarium* species are responsible for numerous diseases in wheat and other small grain cereals cultivated worldwide. *Fusarium culmorum (Wm.G. Sm.) Sacc.* is a threat to plants at every stage of their development. The infection evoked by this pathogen is a serious problem in cereal agriculture. The most common symptoms of Fusarium wilt in wheat include Fusarium seedling blight (FSB), root rot, and Fusarium head blight (FHB). These symptoms have especially disadvantageous effects on plant growth, development, grain yield, and its quality [1,2,3]. The yield reduction is an outcome of damaged kernels which appear discolored and shriveled. *Fusarium culmorum* belongs to the fungi producing numerous dangerous toxins, such as deoxynivalenol (DON) (Figure 1A), nivalenol (NIV) (Figure 1B), T-2 toxin (Figure 1C), and zearalenone (ZEN) (Figure 1D). These mycotoxins represent the trichothecenes family, i.e., epoxy-sesquiterpenoid metabolites responsible for pathogenic virulence and protein synthesis [4,5]. Food products and fodder contaminated with secondary metabolites of *F. culmorum* may evoke severe and chronic harm to human and domestic animal health [6,7,8]. In the food industry, grain infected with *Fusarium,* in which the level of mycotoxins exceeds the permissible EU standards, must be discarded. The maximum limit of toxins are: 750 µg·kg^−1^ DON and 75 µg·kg^−1^ ZEN in flour, and 500 µg·kg^−1^ DON and 50 µg·kg^−1^ ZEN in bread. The toxin levels are also established for feed production at 900 µg·kg^−1^ DON for pigs and 100 µg·kg^−1^ ZEN for piglets [9,10].

Resistance to *Fusarium* head blight is a complex, quantitative trait. Several types (mechanisms) of resistance were identified, and they were described as: Type I—resistance to an initial infection; type II—resistance to the pathogen spread within the host; type III—kernel damage; type IV—tolerance to trichothecene toxins; type V—resistance to toxin accumulation [11,12]. In response to the presence of the pathogen, the host plant activates defense processes, e.g., alters the production of some biochemical components, such as soluble sugars, phenolic compounds, hormones, or reactive oxygen species (ROS) [13]. Sugars play a pivotal role in the immune processes, especially in pathogen attacks, by initiating a signal transduction pathway and regulating the osmotic potential [14,15,16]. Increased concentration of phenolic compounds is toxic to pathogens and prevents further infection. Phenolics are involved in the lignification of the cell wall, which increases the structural barrier that hinders the spread of the pathogen within the host plant tissue. The lignification may reduce the transfer of nutrients from the host plant cell to the pathogen [17]. Due to their toxic nature, phenolic compounds, such as phytoalexins, are considered activators of pathogen resistance genes and modulators of pathogen toxicity [18]. Another way to prevent pathogen infection is a mechanism that involves the production of enzymatic and non-enzymatic antioxidants, and scavenging of reactive oxygen species (ROS) [19]. The ROS includes non-radical molecules, such as hydrogen peroxide (H_2_O_2_) and singlet oxygen (^1^O_2_), as well as free radicals, such as superoxide anion (O_2_^• −^) and hydroxyl radical (OH^•^) [20]. Reactive oxygen species can perform three functions: They can act as cell-damaging agents, signal transduction molecules, and can provide protection against pathogenic microbes [21]. Excessive production of ROS is often called an oxidative burst. Overproduction of ROS can lead to protein and chlorophyll oxidation, damage to nucleic acids, lipid peroxidation, or initiation of programmed cell death [22,23]. Reactive oxygen species accumulation is counteracted by the activation of enzymatic antioxidants, such as catalase (CAT), peroxidase (POX), superoxide dismutase (SOD), and non-enzymatic antioxidants, such as low molecular weight (LMW) phenolics and carotenoids [21,24,25]. Catalase is responsible for the decomposition of H_2_O_2_ into H_2_O and O_2_, as well as for the regulation of H_2_O_2_ concentration in plant tissues. This enzyme is involved in plant development, but also plays an important role in plant resistance to pathogens and aging processes [26]. Peroxidases have a similar function to CAT, as they are involved in scavenging ROS in response to pathogen-plant interactions. In addition, POXs are responsible for the oxidation of phenolics, making them more toxic towards pathogens, lignin biosynthesis, suberization, and growth of the plant cell walls [27]. Superoxide dismutase plays an equally pivotal role in maintaining redox balance and defense response in plants exposed to stress. Its task is to catalyze the dismutation of O_2_^•−^ and HO_2_^•^ (hydroperoxide radical) to H_2_O_2_ and H_2_O. Superoxide dismutase is the first line of defense against a pathogen attack and protects plants from oxidative stress [28]. Hydrogen peroxide also plays a significant role in pathogen defense. Thanks to its antimicrobial properties, it can induce local and systemic resistance to pathogen infection in plants [29].

Pathogen presence can also affect the level of chlorophyll pigments and their activity, resulting in altered efficiency of photosystem II (PS II) [30]. Similar observations were reported by other authors examining the photosynthetic pigment content after *F. culmorum* infection in tomato [31] and barley [32]. The investigated pathogen predominates in cooler areas of northern, central, and western Europe, and it infects wheat, barley, and oats [33]. Grain of durum wheat (*Triticum turgidum* L. subsp. *durum* (Desf.) Husn.) is used primarily in the production of pasta and to a lesser extent in the production of bread and groats. Although, durum wheat originates from the Mediterranean region and the countries of the Middle East is also very sensitive to *F. culmorum* [34]. Recent years have brought increased interest in durum wheat cultivation in Poland. Major problems with this crop include its high sensitivity to drought, soil salinity, cadmium accumulation, and *Fusarium* infections [34,35,36,37]. Durum wheat, as compared with common wheat (*T. aestivum*), is characterized by higher sensitivity to *Fusarium* infection. This is attributed to its morphological traits, such as early flowering, longer awn, another retention inside the floret, spike compactness, and genetic differences, such as the presence of type I rather than type II resistance genes [38,39]. 

In the presented study, three durum wheat accessions were assessed in terms of resistance to *Fusarium* diseases at two stages of their ontogenesis: Two-week-old seedlings and full anthesis stage—65 BBCH scale [40]. The defense response of the studied durum genotypes included evaluation of the resistance degree in the seedlings by means of visual inspection of the leaves and roots, and fresh weight measurements. We also determined the content of total soluble carbohydrates, total soluble phenolics and cell wall-bound phenolics, chlorophyll pigments, hydrogen peroxide, and antioxidant enzymes activity. At the full anthesis stage, we visually evaluated the resistance to *Fusarium* head blight, and measured the content of mycotoxins (deoxynivalenol, nivalenol, T–2 toxin, and zearalenone), and yield parameters. The main objective of the study was to identify physiological or biochemical markers of resistance to *F. culmorum* at both developmental stages in three durum wheat accessions. The investigation was carried out on Polish line SMH87 and two Australian accessions: cv. ‘Tamaroi’ and BC_5_Nax_2_ line. The selected genotypes differed in the degree of resistance to salinity and were the subject of our earlier studies on cadmium accumulation in the grain of durum wheat [36,37].

## 2. Results

### 2.1. Experiment I

#### 2.1.1. Disease Rating (DR) and Fresh Weight (FW) Loss

The visual disease rating (DR) included only the seedlings infected with *F. culmorum*, so control seedlings were not evaluated. In this experiment, the vigor of plant seedlings at the three-leaf stage was assessed. All analyses, described in Section 2.1., were done on material collected in this developmental stage. The leaves and roots of SMH87 line and cv. ‘Tamaroi’ showed higher sensitivity to the pathogen infection than those of the BC_5_Nax_2_ line (Figure 2). The highest percentage of leaf DR was observed in SMH87 plants, while the lowest in the BC_5_Nax_2_ line. Root infection results were similar in SMH87 and cv. ‘Tamaroi’. Roots of BC_5_Nax_2_ were infected to the same degree as in cv. ‘Tamaroi’, but lower than in SMH87.

**Figure 3:**
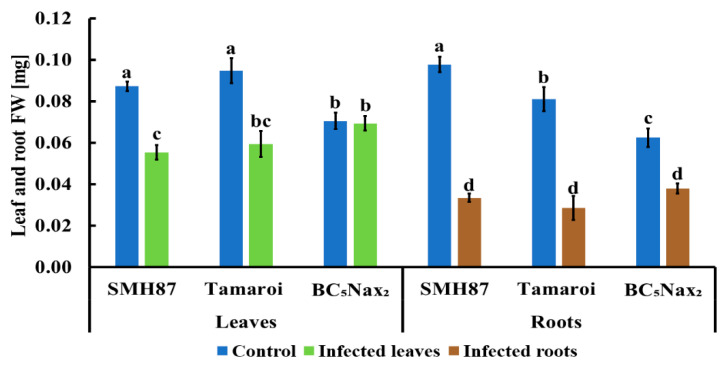
Effects of *F. culmorum* infection on fresh weight (FW) of the leaves and roots of three durum wheat accessions. The values represent means (*n* = 30) ± SE. Different superscript letters (a–d) for each organ indicate significant differences between means (Duncan’s multiple range test; *p* < 0.05).

*Fusarium culmorum* infection negatively affected leaf and root FW in all studied accessions (Figure 3). The effects were more pronounced in the roots than in the leaves. In SMH87 and cv. ‘Tamaroi’ FW reduction of the infected leaves was greater than in BC_5_Nax_2_. *Fusarium* infection caused a 37% decrease in leaf FW in both SMH87 and cv. ‘Tamaroi’ as compared with control. The infected roots of SMH87 and cv. ‘Tamaroi’ demonstrated a 66% decrease in FW in comparison with control. BC_5_Nax_2_ line did not show FW reduction in the infected leaves, but in the infected roots, FW loss amounted to 39% versus that of control.

#### 2.1.2. Chlorophyll *a, b,* and Carotenoid (Chl*a, b*, and car) Content

**Figure 4:**
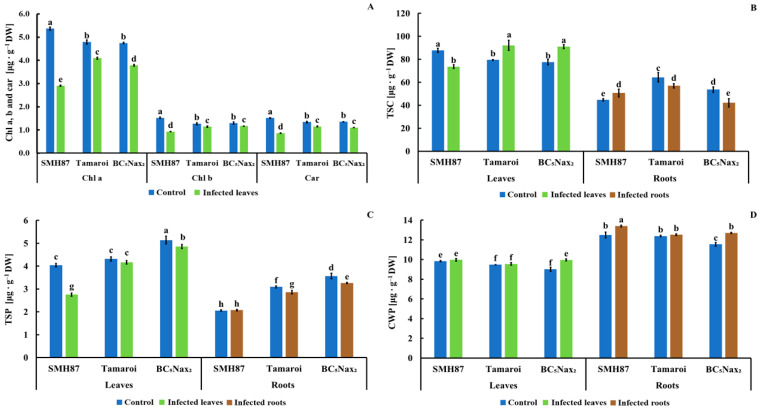
Effect of *F. culmorum* infection on the content of (**A**) chlorophyll *a*, *b* (Chl*a*, *b*) and carotenoids (Car), (B) total water-soluble carbohydrates (TSC), (**C**) total soluble phenolics (TSP), (**D**) cell wall-bound phenolics (CWP) in the leaves and roots of three durum wheat accessions. The values represent means (*n* = 30) ± SE. Different superscript letters (a–h) for each organ indicate significant differences between means (Duncan’s multiple range test; *p* < 0.05).

The infection reduced the contents of chlorophyll *a*, *b*, and carotenoids (Chl*a, b*, and car) in all studied durum wheat accessions (Figure 4A). The greatest decrease in all pigments was observed in SMH87 leaves. Chlorophyll *a* content dropped by 48%, Chl*b* by 40%, and carotenoids by 44% as compared with control plants. The other studied accessions also showed pigment content reduction in the infected plants; however, these differences, although significant, were not as drastic as in the SMH87 line.

#### 2.1.3. Total Soluble Carbohydrates (TSC)

As a result of the infection, TSC content in SMH87 plants (Figure 4B) decreased in the leaves and increased in the roots, as compared with control. An opposite trend was observed in cv. ‘Tamaroi’ and BC_5_Nax_2_, where the infection triggered an increase in TSC in the leaves and a drop in the roots as compared with control.

#### 2.1.4. Total Soluble Phenolics (TSP)

The studied genotypes differed in the content of total soluble phenolics (TSP) in the control and infected leaves and roots (Figure 4C). Line SMH87 showed a significant, almost 32% decrease of TSP content in the infected leaves, but no changes in the roots. In the infected leaves and roots of BC_5_Nax_2_ a reduction in TSP was observed, while in cv. ‘Tamaroi’ the infection decreased phenolic content in the roots. Total soluble phenolics levels in the leaves of this cultivar were unaffected by the infection. 

#### 2.1.5. Cell Wall-Bound Phenolics (CWP)

*Fusarium* infection boosted CWP only in the leaves of BC_5_Nax_2_ (Figure 4D). In the roots of SMH87 and BC_5_Nax_2_, an increase in CWP was observed upon inoculation. In other cases, the infection did not cause significant changes in the content of these compounds.

#### 2.1.6. Catalase (CAT) Activity

**Figure 5:**
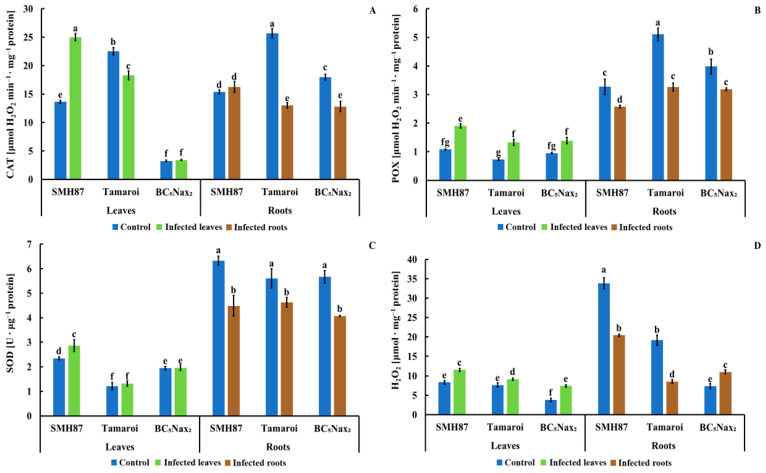
Effects of *F. culmorum* infection on catalase (**A**), peroxidases (**B**), and superoxide dismutase (**C**) activity, and H_2_O_2_ (**D**) content in the leaves and roots of three durum wheat accessions. The values represent means (*n* = 3) ± SE. Different superscript letters (**a–g**) for each organ indicate significant differences between means (Duncan’s multiple range test; *p* < 0.05).

The greatest increase in CAT activity is due to the infection was observed in SMH87 leaves. In cv. ‘Tamaroi’, it was diminished, and in the BC_5_Nax_2_ line, it was unaffected by the infection (Figure 5A). Catalase activity was considerably higher in control roots of cv. ‘Tamaroi’ and BC_5_Nax_2_ than in the infected ones. There was no difference in CAT activity in the infected and control SMH87 roots.

#### 2.1.7. Peroxidases (POXs) Activity

An increase in POXs activity was observed in the leaves of all infected genotypes (Figure 5B). The leaves of SMH87 and cv. ‘Tamaroi’ showed a 44% increase in POXs activity as compared with control, while in BC_5_Nax_2_ leaves, this increase amounted to 31%. In the roots ofSMH87, ‘Tamaroi’, and BC_5_Nax_2_, the infection brought about a significant decrease in POXs activity by 23, 35, and 19%, respectively.

#### 2.1.8. Superoxide Dismutase (SOD) Activity

Superoxide dismutase activity was significantly higher only in the infected leaves of the SMH87 line as an effect of *Fusarium* infection (Figure 5C). In the case of other accessions, the infection did not change SOD activity. In the infected roots of SMH87, cv. ‘Tamaroi’, and BC_5_Nax_2_ the infection decreased activity of this enzyme by 30%, 18%, 28%, respectively. 

#### 2.1.9. Hydrogen Peroxide (H_2_O_2_) Content

Hydrogen peroxide content increased in the leaves of all studied accessions as an effect of infection (Figure 5D). The highest increase in H_2_O_2_ level was noted in the infected leaves of BC_5_Nax_2_ plants. A rapid decrease in H_2_O_2_ content was noted in the infected roots of SMH87 (39%) and cv. ‘Tamaroi’ (55%), while in BC_5_Nax_2_ plants, the infection enhanced H_2_O_2_ level in the roots by 33% vs. control.

### 2.2. Correlation Analysis

The disease symptoms assessed with the visual disease rating (DR) negatively correlated with Chl*a*, *b*, and Car content, and with FW of the leaves and roots (Table 1).

Significant correlations between H_2_O_2_ content and the investigated enzymes were found (Table 2).

In the leaves, CAT and POX positively correlated with H_2_O_2_ content. This relationship was not observed in the roots. Superoxide dismutase activity correlated positively with H_2_O_2_ only in the roots (r = 0.613, *p* < 0.05), proving that the higher SOD activity, the more H_2_O_2_ was produced. In the leaves, TPC content negatively correlated with H_2_O_2_ levels, while TPC content in the roots negatively correlated with H_2_O_2_ concentration in both studied organs (Table 2.). Significant correlation between TSP and TSC (r = 0.63118, *p* < 0.05) was found.

### 2.3. Experiment II

#### 2.3.1. Fusarium Head Blight Index (FHBi) and Yield Parameters

Index FHBi showed a significant difference between the studied genotypes regarding *F. culmorum* resistance (Table 3). The time after infection on the spikes significantly impacted the disease development. The research revealed the fastest and the strongest spike infection (10%) in the SMH87 line seven days after the first inoculation. After 14 days, a 71% increase in FHBi of SMH87 spikes vs. the first evaluation was observed. The spike inoculation with *Fusarium* spores significantly reduced the amount of grain per spike in all studied genotypes (Table 3). A significantly higher grain reduction occurred in SMH87, where it was 83% greater than in control. A strong reduction in grain number (64%) was also observed in cv. ‘Tamaroi’.

BC_5_Nax_2_ line showed the lowest reduction of grain yield per spike in the inoculated plants. The analysis of yield parameters showed significant differences in grain mass per spike and in the mass of one piece of grain, as well as MTS of the control and inoculated plants (Table 3). A significantly higher (83%) reduction of grain mass per spike was observed in SMH87 plants. BC_5_Nax_2_ plants showed a significantly lower (by 40%) reduction in grain mass per spike as compared with the other studied accessions. Analyses of the mass of one piece of grain and of one thousand grains revealed significant differences between the studied accessions (Table 3). A significant loss of a single grain mass was observed in the SMH87 line, dropping by 75% vs. control plants. A 45% reduction in MTS was observed in BC_5_Nax_2_ plants. This genotype showed the lowest MTS reduction, while the highest MTS reduction was seen in SMH87. 

#### 2.3.2. Mycotoxin Content

Trace amounts of DON and ZEN were found in the grain of all control plants, while the concentration of T–2 was ten times higher (Table 4). The amount of NIV in control plants of ‘Tamaroi’ and BC_5_Nax_2_ was four times higher than that in SMH87. The infection considerably increased the levels of NIV, DON, and T–2 toxins. The highest amount of NIV was recorded in cv. ‘Tamaroi’ grain after inoculation and it was six times higher than that of the control. In the grain of the infected SMH87, the level of this toxin was 18 times higher than in control, while in the infected BC_5_Nax_2_ plants—four times higher. In BC_5_Nax_2_ grain, NIV level after inoculation increased four times as compared with control. As in the case of NIV, cv. ‘Tamaroi’ grain contained the highest level of DON. The grain content of ZEN was the lowest among the other studied toxins. Inoculation did not increase ZEN content in SMH87 seeds, while in cv. ‘Tamaroi’ and BC_5_Nax_2_, the level of this toxin was more than six times higher than in the control. In all cases, inoculation slightly increased the content of T–2 in the seeds as compared with that of the control. The highest increase in T–2 was found in cv. ‘Tamaroi’ (2.3×), while in SMH87 and BC_5_Nax_2_, the increase was 1.64 and 1.75×, respectively. To summarize, cv. ‘Tamaroi’ showed the highest toxin accumulation in grain. 

#### 2.3.3. Correlation Analysis

*Fusarium* head blight index (FHBi) evaluated 7 and 14 days after inoculation negatively correlated with the number of seeds and their mass (Table 5). The content of NIV and DON significantly decreased all studied yield parameters, while ZEN reduced only the mass of a single grain. No correlation between the content of T–2 and the evaluated yield parameters was found. The latter two results can be explained by low content of both toxins.

Strong correlation was detected between the visual assessment of *Fusarium* head blight index (FHBi) evaluated in both terms (7 and 14 days after infection) and the grain content of all investigated mycotoxins, except for T–2 (Table 6). Index FHBi positively correlated with the content of DON and NIV, while in the case of ZEN, a significant correlation was found only seven days after the infection.

## 3. Discussion

*Fusarium culmorum* attacks plants at various developmental stages. The pathogenesis is responsible for the formation of seedling blight and root rot, which limit seedling emergence and plant development [32]. In our experiment, the infection caused a darkening of the roots and slower leaf growth. The studied genotypes differed more in the degree of leaf than root infestation. SMH87 line was the most, and BC_5_Nax_2_the least heavily infested. Medium infestation degree was observed in cv. ‘Tamaroi’. This result was surprising, since the genotypes originating from a much warmer and drier climate were less severely infected than the original genotype from Poland. The infection degree was visible in leaf FW loss: BC_5_Nax_2_ genotype did not show changes in fresh leaf weight, while the decrease in root weight, although significant, was the smallest among the studied genotypes. Similar results were obtained by Grey and Mathre [41] in barley, by Wojciechowski et al. [42] in winter wheat, and by Warzecha et al. [43] in oats. These authors suggest that the most severe damage caused by *Fusarium* seedling blight appeared in the roots. It indicates that visual evaluation of root infestation may be more useful than leaf assessment. According to Malalaseker et al. [44] and Knudsen et al. [45], root rot may also develop, due to a prior infestation of hypocotyls and shoots. Root infection negatively affects proper plant development and disturbs basic physiological processes, such as distribution of assimilates, water uptake and transport, and soil mineral absorption. These disturbances result in reduced seedling vigor and interrupted growth which negatively affects grain quality and yield.

Fungal mycelium penetrates the host-plant cells and limits access to nutrients and water. Released toxins disrupt metabolic and physiological processes. This leads to the reduction of photosynthetic pigment content and disturbances of photosynthesis [44]. Our study demonstrated that *F. culmorum* infection significantly decreased the content of chlorophyll *a*, *b*, and carotenoids in the leaves. Similar observations were published by other researchers examining the content of photosynthetic pigments after *F. culmorum* infection in tomato [31] or barley [32]. In our study, the results of visual assessment of DR in the leaves and roots negatively correlated with the content of Chl*a*, *b*, and Car.

Soluble sugars play an important role in plant development and metabolism, and therefore, their content fluctuates during plant infection. Soluble sugars in the host-plant cells are a source of carbon for the pathogen [46,47,48]. Sucrose was shown to induce defense mechanisms in the infected cells. The hexose, through signal transduction by hexokinase, increases the production of peroxidases and proteins directly related to pathogenesis [14,16]. Soluble sugars, as compounds with higher osmotic potential, limit the spread of the infection. Moreover, they isolate healthy cells from the infected ones and protect them against water loss [49]. Our analyzes of TSC showed that infection significantly increased TSC content in cv. ‘Tamaroi’ and BC_5_Nax_2_ leaves and decreased TSC levels in the roots. A contrary trend was observed in the leaves and roots of SMH87 line. Warzecha et al. [32] noted an increased sugar content in the leaves and their decrease in the roots of barley infected by *F. culmorum*. Morkunas et al. [16] reported that increased content of soluble sugars supported the resistance of *Lupinus luteus* L. to *F. oxysporum* infection, while Gaudet et al. [15] observed a similar correlation in wheat infested by snow mold fungi. Bani et al. [50] suggested that *Fusarium* species infection during seed germination disrupted sugar distribution between cotyledons and the tissues of embryo axis in the germinating seeds. Formela-Luboińska et al. [51] reported that soluble carbohydrates reduced sporulation of *F. oxysporum* f. sp. *lupini* and limited the production of moniliformin toxin synthesized by this *Fusarium* species. In our study, the more resistant Australian accessions (cv. ‘Tamaroi’, BC_5_Nax_2_) showed higher sugar content in the leaves of infected seedlings than Polish SMH87. Our research demonstrated that sugar content in the leaves was a stronger indicator of *F. culmorum* resistance than that in the roots. 

Synthesis of phenolic compounds is a well-known defense response to pathogen attack. Their biosynthesis occurs both before and after the infection [52]. The defensive role of phenolics in fungal infections in plants was confirmed in our previous studies [53,54,55]. The phenolic compounds involved in the immune response to pathogen attack include salicylic and chlorogenic acids. Salicylic acid controls the content of the signal molecule hydrogen peroxide (H_2_O_2_) responsible for plant resistance to environmental stresses. Salicylic acid activates superoxide dismutase (SOD), which boosts H_2_O_2_ production and stimulates the synthesis of pathogenesis related proteins (PR)—chitinases and glucanases that decompose the cell wall of the fungal hyphae [56,57]. Salicylic acid participates in systemic acquired resistance (SAR). This reaction is triggered in the case of biotrophic fungi infection. The fungi from *Fusarium* species are classified as hemibiotrophic ones, which means that the pathogens initially behave like biotrophic fungi and then switch on to the optional parasitization mode [52]. Another group of compounds participating in the immune response to pathogens is phytoalexins, i.e., low molecular weight phenolics. They are derivatives of benzoic acid, stilbene, coumarin or quercetin [58,59]. The synthesis of phenolic compounds requires a large energy input, and therefore, it depends on the accumulation of the number of soluble sugars in the cells. We confirmed this correlation in our experiments. High correlation (r = 0.631; *p* < 0.05) between TSC and TPC may indicate the plant defense response to the infection consisting in the increase of TSC consumption for ATP synthesis and further use of this energy in the synthesis of phenolics. SMH87 plants, more sensitive to *F. culmorum*, showed a significant decrease in the phenolic content in the leaves as compared with the other accessions. Contrary to that, the most resistant BC_5_Nax_2_line was characterized by the highest content of phenolics in the leaves and roots of the control and infected seedlings. Hakulinen et al. [60] suggested that the lowered content of phenolics may be caused by the synthesis of lignin that is a polymer of oxidized phenolic alcohols. Lignin fortifies cell walls making them difficult for fungal hyphae to colonizing the host plant [61,62]. Datta and Lal [63] and Noman et al. [64] reported this phenomenon as a hypersensitivity reaction initiated as a plant defense mechanism to developing an infection. In our experiment, a decrease in leaf TPC was associated with higher content of cell-wall-bound phenolic compounds (CWP) only in BC_5_Nax_2_. The same line revealed a relationship between decreased root TPC and increased accumulation of CWP. The leaf CWP content positively correlated with the content of H_2_O_2_ (r = 0.679; *p* < 0.05). In the leaves, TPC content negatively correlated with H_2_O_2_ levels, while TPC content in the roots negatively correlated with H_2_O_2_ concentration in both studied organs. These negative correlations may suggest that during *Fusarium* infection, TPC acted as antioxidants and possibly reduced H_2_O_2_ amount.

Antioxidant enzymes, such as CAT, POXs, and SOD, form the first line of defense against ROS during the entire pathogenesis [65,66,67]. Superoxide dismutase (SOD) is responsible for the dismutation of the superoxide radicals to molecular oxygen and hydrogen peroxide. CAT and POX decompose H_2_O_2_. Some studies reported that *Fusarium* infections boosted the activity of the antioxidant enzymes [68,69,70]. In the investigated wheat seedlings, we detected greater POX and SOD activity in the roots than in the leaves. It can be explained by the fact that the in vitro infection started in the roots growing in the infected medium. In the roots we observed a correlation between SOD activity and H_2_O_2_ accumulation (r = 0.613; *p* < 0.05), while in the leaves there was a correlation between CAT and POX activity and H_2_O_2_ (r = 0.710 and r = 0.688; *p* < 0.05, respectively). We recorded high negative correlation between TPC content and CAT and POX activity in the leaves (r = −0.788 and r = −0.515; *p* < 0.05, respectively). These results may suggest a competition between antioxidant enzymes and phenolic compounds for H_2_O_2_, which can indicate the antioxidant properties of phenolics. We reported higher activity of the antioxidant enzymes and higher levels of H_2_O_2_ in the control leaves than in the control roots. The infection decreased the activity of the antioxidant system in the roots, but not in the leaves. The enzyme activity poorly differentiated the studied accessions regarding their resistance to *F. culmorum.* Only CAT activity was twofold higher in the infected SMH87 leaves, considered by us to be more sensitive to *Fusarium*, while in cv. ‘Tamaroi’ and BC_5_Nax_2_ line this activity was lower or remained unchanged. Płażek and Żur [71] indicated that low activity of CAT could be a marker of a plant resistance to a fungal infection, as CAT decomposes H_2_O_2_ that is necessary for the defense as a signal molecule. 

Fusarium head blight causes huge yield losses in cereals, reaching over 40%. The disease reduces grain yield, its mass, nutritional value and leads to grain contamination with mycotoxins [6]. Our research confirmed that spike infection not only reduces the grain mass, but also lowers the final yield. The reduction of yield parameters is also associated with high concentrations of mycotoxins in the grain. Negative correlation between the examined yield parameters (amount of grain per spike, mass of grain per spike, mass of a single grain, and mass of thousand seeds) and the content of NIV and DON suggest the reduction of the yield is mainly, due to accumulated toxins. ZEN content only affected the mass of a single grain, and we found no relationships between T–2 toxin and the yield. The visual assessment of spike infestation degree (FHBi) was performed at two terms: Seven and fourteen days after infection. In both terms, FHBi highly negatively correlated with the yield parameters. It could be stated that FHBi, especially 7 days after the infection, is a reliable method to determine cereal resistance to the infection, as confirmed by other studies [72,73].The statistical analysis showed a strong correlation between FHBi 7 and 14 days after the infection, and DON accumulation in the grain (r = 0.733, r = 0.632, *p* < 0.05, respectively), and NIV content (r = 0.731, r = 0.630, *p* < 0.05, respectively). A similar relationship between FHBi and DON accumulation was observed by Haidukowski et al. [74] in common wheat. Nowicki et al. [75] and Pascale et al. [76] claimed that FHBi can be used to predict the grain contamination degree with mycotoxins before performing detailed analyses. In our research, we used NIV-chemotype isolate of *F. culmorum,* which is considered a milder *Fusarium* chemotype than DON-chemotype or acetyl derivatives (3AcDON, 15AcDON) [77,78]. Desjardin and Plattner [79] reported that *F. culmorum* NIV-chemotypes can produce DON, but in amounts <1% of NIV, while DON-chemotypes are not capable of producing NIV [80]. In our experiments, we observed increased level of DON in relation to NIV, which contradicted the hypothesis presented by Dejsrdin and Plattner [79].

## 4. Materials and Methods

### 4.1. Plant Material and Experimental Design

Two experiments were performed in 2020. The first was an in vitro assay testing the plant resistance to *Fusarium*, and the other involved plants at the anthesis stage grown in an open foil tunnel. Three durum wheat accessions differing in salt–stress tolerance were used: Polish moderately sensitive SMH87 line (courtesy of Dr. Jarosław Bojarczuk, Plant Breeding Centre in Smolice, IHAR Group, Poland), sensitive Australian cultivar ‘Tamaroi’, and resistant BC_5_Nax_2_ line (courtesy of Dr. Richard A. James, CSIRO Plant Industry, Acton, Australia). These accessions were investigated in previous studies on durum wheat tolerance to salinity and cadmium. 

#### 4.1.1. Preparation of *Fusarium culmorum* Isolate

In both experiments, the plants or seeds were infected with IPO348–01 nivalenol chemotype mycelium of *F. culmorum* from the Plant Breeding Institute, Wageningen (Netherland). Mycelium box test was performed in in vitro conditions on seedlings grown on inoculated Potato Dextrose Agar (PDA) medium (Sigma–Aldrich, Poznań, Poland). The mycelium was grown in a microbiological thermostatic chamber (ST 5 Smart, Pol–Aura Aparatura, Wodzisław Śląski, Poland) at 21 °C, in darkness for seven days [32]. The mycelium production for the open tunnel experiment followed the method described by Wiśniewska et al. [81]. An Erlenmeyer glass flask (250 cm^3^) was filled with 50 g of spring wheat seeds, and 15 cm^3^ of water w added to obtain 40% humidity. After 24 h, the seeds were autoclaved at 101 325 Pa, at 121 °C for 30 min and then cooled. The infection was initiated by transferring three 1.5 cm discs of PDA medium. The glass flasks were placed in the microbiological thermostatic chamber (ST 5 Smart, Pol–Aura Aparatura, Wodzisław Śląski, Poland), and the mycelium was growing at 20 ± 1 °C for five to six weeks in darkness. The flasks were shaken thoroughly every day to prevent sticking the grain to the glass, and to also provide uniform inoculation of the grain.

#### 4.1.2. Experiment I—Box Test Assay

**Figure 6:**
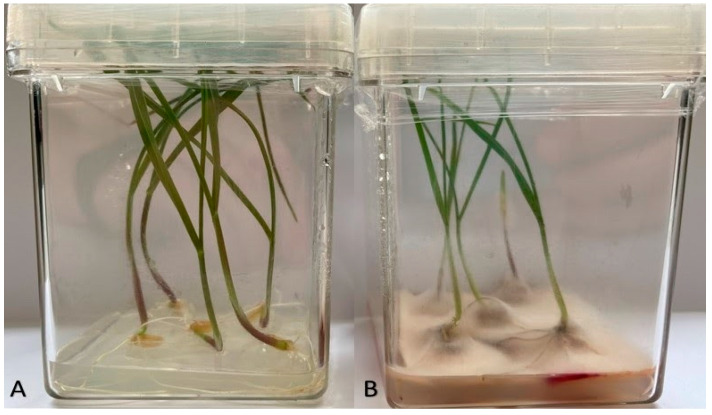
Two-week old durum wheat seedlings growing in pathogen-free medium (**A**) and the medium infected with *F. culmorum* medium (**B**).

This experiment was performed on the seedlings grown in Magenta GA–7 Boxes (Sigma–Aldrich, Poznań, Poland) under sterile conditions. The boxes were filled with 20 cm^3^ of MS medium [82]. Discs (5 mm) of PDA medium overgrown with *Fusarium* mycelium were cut and transferred into magenta boxes on MS medium (five discs per box). The seeds were surface disinfected in 20% commercial bleach (active ingredient sodium hypochlorite) for 20 min, rinsed three times for 2 min with sterile water, and transferred into Petri dishes lined with wetted sterile filter paper for 24 h germination. The germinating seeds (five seeds per Magenta-Box) were placed on mycelium discs. The control seeds were placed on PDA medium discs free of the pathogen (Figure 6). The experiment was performed in six replicates for each accession/treatment (magenta with control and inoculated seeds) combination. Vegetation in both treatments was conducted on MS medium. Vegetation conditions were maintained for 14 days in a growth chamber at 22/20 °C (day/night), the light intensity of 150 μmol m^−2^·s^−1^ PPFD (Photosynthetic Photon Flux Density) and 12 h/12 h (day/night) photoperiod with 100% air humidity.

Two weeks after inoculation, the leaves and roots were collected separately, weighed, and frozen in liquid nitrogen. The subsequent analyses involved: Visual disease rating (DR), chlorophyll *a*, *b*, and carotenoid content (Chl*a*, *b*, and car), total soluble carbohydrate content (TSC), the content of total phenolic compounds (TPC), and cell wall-bound phenolics (CWP), antioxidant enzyme activities, hydrogen peroxide content (H_2_O_2_), and fresh weight (FW) of leaves and roots. 

#### 4.1.3. Experiment II—Open Foil Tunnel

The experiment was carried out in semi-controlled conditions in an open foil tunnel. The seeds were sown into plastic pots (20 × 20 × 25 cm; nine seeds per pot), in six replicates (six pots) for each accession/treatment (control and inoculated plants) combination. The plants were cultivated in universal garden soil substrate pH = 5.8 (Ekoziem, Jurkow, Poland) mixed with sand (1:1, *v*/*v*). Before sowing, the seeds were sterilized in 70% ethanol for one minute and rinsed with sterile water three times for two minutes. Once a week, the plants were fertilized with Hoagland medium [83]. The plants were cultivated until the full anthesis stage—65 BBCH scale [40]. Their spikes were sprayed with the inoculum containing *F. culmorum* spores, while control spikes were sprayed with distilled water. Disease symptoms were evaluated seven and fourteen days after inoculation (DAI 7 and DAI 14). Ripe seeds were collected, and the following yield parameters were evaluated: Number and weight of seeds per spike, the weight of a single seed, and weight of one thousand seeds. Moreover, the content of the following mycotoxins was determined: deoxynivalenol (DON) and its derivatives 3–acetyldeoxynivalenol (3AcDON), 15–acetyldeoxynivalenol (15AcDON), nivalenol (NIV), zearalenone (ZEN) and its derivatives alpha–Zearalanol (α–ZAL), beta–Zearalanol (β–ZAL), alpha–Zearalanol (α–ZEL), beta–Zearalenol (β–ZEL), and T–2 toxin.

### 4.2. Analyses

#### 4.2.1. Disease Rating (DR) and Loss of Fresh Weight (FW)

Direct assessment based on disease rating (DR) was calculated with the formula described by Warzecha et al. [84] to determine the effect of infection on seedling and root development.

DR% = 100 × (n*_i_* × D*_i_*)/ND_max_

where: n*_i_*—number of plants of *i*th category, D*_i_*—numerical value of *i*th category, N—total number of plants in the sample, and D_max_—maximum scale value (0–5) [42]. 

To assess the impact of the infection, we also determined the fresh weight (FW) of leaves and roots. All measurements were done in thirty replicates for each cultivar/line.

#### 4.2.2. Chlorophyll (a, b) and Carotenoid (Car) Content

Chlorophylls and carotenoids were estimated spectrophotometrically, according to Czyczyło-Mysza et al. [85]. Plant Leaves samples were dried at 65 °C for 48 h, weighed, and then extracted in 96% ethanol (5 mg/1.5 cm^3^), and centrifuged (21,000× *g*, 5 min at 15 °C). The extract was transferred to 96 well plate, and the absorbance was read at 470, 648, and 664 nm (Synergy II, Biotek, Winooski, VT, USA). The concentrations of Chl*a*, Chl*b*, and total carotenoids (car) were calculated using Lichtenthaler and Buschman [86] equations:

Chl*a* (μg/cm^3^) = 13.36 A_664_ − 5.19 A_648_

Chl*b* (μg/cm^3^) = 27.43 A_648_ − 8.12 A_664_

Car (μg/cm^3^) = (1000 A_470_−2.13 Chl*a* − 97.64 Chl*b*)/209

where: Chl*a* = chlorophyll *a,* Chl*b* = chlorophyll *b,* A_470_ = absorbance at 470 nm, A_664_ = absorbance at 664 nm, A_648_ = absorbance at 648 nm.

#### 4.2.3. Determination of Total Water-Soluble Carbohydrates (TSC)

Sugars were analyzed by the phenol-sulfuric method of Dubois et al. [87], with modifications reported by Bach et al. [88]. The samples extracted, as for pigment estimation (10 µL) were diluted to 200 μL with water, and 200 μL of 5% phenol (*w*/*w*) solution was added. Then, 1 cm^3^ of concentrated sulfuric acid was dispensed, the samples were mixed, and after 20 min incubation at ambient temperature and transferred to 96-well plates and absorbance was read at 490 nm (Synergy II, Biotek, Winooski, VT, USA). Sugar content was expressed as glucose equivalents—using the calibration curve obtained with a standard solution of glucose.

#### 4.2.4. Determination of Total Phenolic Compounds (TPC)

Estimation of total phenolic content was done according to the Singleton method with modifications [88]. The extracts (prepared as described for pigments) were mixed with water diluted Folin–Ciocalteu phenol reagent (5:2, *v*/*v*) and after 10 min saturated Na_2_CO_3_ (c.a. 25% *w*/*w*) was added (100/400/400 µL). The samples were then incubated for 2 h in darkness, at room temperature. After centrifugation (21,000× *g*, for 15 min at 15 °C), they were transferred to 96-well plates. Their absorbance was recorded at 760 nm (Synergy II, Biotek, Winooski, VT, USA). The pool of phenolic compounds was expressed as mg of gallic acid—using the calibration curve obtained with a standard solution of gallic acid.

#### 4.2.5. Determination of Cell Wall-Bound Phenolics (CWP)

The pellets remaining after extraction of pigments were rinsed with ethanol and hydrolyzed with 3 M NaOH [89] overnight, at room temperature. Then the samples were neutralized with concentrated HCl, then ethanol was added (1 cm^3^ per sample), and the resulting solution was analyzed for released phenolics as for soluble forms described in Section 4.2.4. 

#### 4.2.6. Activity of Enzymatic Antioxidants

Plant material was homogenized at 4 °C in 50 mM (pH 7) phosphate-potassium buffer containing 0.1 mM EDTA (100 mg of FW plant material per 1 cm^3^ of buffer). The activity of superoxide dismutase (SOD, EC 1.15.1.1), catalase (CAT, E.C. 1.11.1.6), and peroxidase (POX, EC 1.11.1.7) were determined. After centrifugation (10,000× *g*, 15 min at 4 °C, 32R, Hettich, Germany), clear supernatant was sub-sampled and assayed for SOD, CAT, and POX activity in 96-well plate format (Synergy II, Biotek, Winooski, VT). SOD activity was determined by the cytochrome reduction method of McCord and Fridovic [90]. CAT activity was measured at 240 nm according to Aebi [91], with H_2_O_2_ as a substrate. The activity of POX was assessed using the Lück [92] method with *p*–phenylenediamine as substrate, the absorbance was monitored at 485 nm. The analyses were conducted as described by Gudys et al. and references are cited therein [93,94,95]. Enzyme activities were presented on a protein basis. Protein content was assayed with the standard Bradford method [96].

#### 4.2.7. Hydrogen Peroxide Content

Plant material was homogenized at 4 °C in 50 mM (pH 7) phosphate-potassium buffer containing 0.1 mM EDTA, as described for enzyme activity analyses. Hydrogen peroxide content was estimated with a commercial Amplex Red (10–acetyl–3,7–dihydroxyphenoxazine) [97] reagent kit (Invitrogen, Waltham, MA, USA), according to the manufacturer’s manual [98]. Briefly, the plant sample was diluted with the reaction buffer, and the working solution containing fluorescence probe precursor (Amplex Red), and 0.2 U·cm^−3^ horseradish peroxidase was added. After 30 min incubation, fluorescence was read at Ex/Em 530/590 nm in 96-well plate format (Synergy II, Biotek, Winooski, VT, USA). The results were quantitated based on a calibration curve made for H_2_O_2_.

#### 4.2.8. Spore Suspension Preparation and Spike Inoculation Procedure

The spikes were inoculated with the spore suspension of *F. culmorum* prepared as described by Góral et al. [12]. The grain inoculated with *F. culmorum* mycelium and conidia were soaked in distilled water for 1 h and then filtered over two layers of sterile cheesecloth. The spore concentration of the suspension was adjusted to 5 × 10^5^ spores · cm^−3^ using Thoma’s chamber. The inoculation was performed according to the methodology described by Warzecha et al. [99], with slight modifications. The spikes from each line were sprayed separately with a hand sprayer, using 2 cm^3^ of the conidia suspension per spike, and covered for 48 h with plastic bags. Control plants were sprayed with distilled water, and covered with plastics bags to provide the same experimental conditions. Inoculation was done early in the morning, when the air humidity was relatively high (70–80 %) and the temperature was low (10–14 °C). 

The spike inoculation procedure was performed twice. The first inoculation was done three days before the full anthesis stage and repeated seven days later. Seven days after each inoculation, *Fusarium* head blight index (FHBi) was visually evaluated for each accession and calculated using the formula described by Góral et al. [12]:

FHBi = % of head infection × % of head infection per accession/100

Forty-five spikes from each accession at the full ripening stage were harvested, evaluated for yield reduction after *Fusarium* inoculation, and compared with non-inoculated plants. The following yield parameters were calculated: Amount of grain per spike, grain mass per spike, mass of a single grain, and mass of one thousand grains (MTS). After the evaluation of the yield parameters, seed material was collected and stored at −20 °C until mycotoxin analyzes. 

#### 4.2.9. UHPLC-MS/MS Estimation of Mycotoxin Accumulation

The samples were analyzed for the content of 10 different mycotoxins by using UHPLC–MS/MS (ultrahigh-performance liquid chromatography coupled with tandem mass spectrometer) as reported by Dziurka et al. [100], with modifications. Plant materials were extracted according to the procedure described by Klötzel and Lauber [101]. The ground samples (0.1 g) were extracted three times in 1 cm^3^ of acetonitrile and water (80:20, *v*/*v*) solution (5 min, 30 Hz, 400 MM, Retch, Haan, Germany). Fifty nanograms of heavy-labeled internal standard ([^13^C_18_]–ZEN, and [^13^C_15_]–DON) were added to each sample. After centrifugation, the samples were cleaned up on Bond Elut Mycotoxin cartridges (3 cm^3^ 500 mg, Agilent Technologies, Germany). The column eluate was evaporated under N_2_, and the residue was resuspended in 100 μL of acetonitrile/water (50:50, *v*/*v*), and analyzed. The mycotoxins (DON, NIV, sum of 3–acetyl–DON and 15–acetyl–DON, T–2, sum of zearalenone and its derivatives: a–, b–ZEL, a–, b–ZAN and ZEN, and OTA) were determined using the UHPLC system (Infinity 1260, Agilent Technologies, Germany) with a tandem quadrupole mass spectrometer (QQQ 6410, Agilent Technologies, USA). The samples were separated on a Poroshell 120 Phenyl–Hexyl 2.1 × 5 mm, 2.7 μM column with a gradient of water (A) and methanol (B) both with 0.1% formic acid, from 5% to 75% methanol in 7.5 min, at a flow rate of 0.5 cm^3^ min^−1^. Multiple reaction monitoring (MRM) transitions after positive ESI ionization were used for identification and quantification (details are given in Table S1). Quantitation was based on calibration curves obtained with authentic standards taking account of the recovery rates of the internal standard used. The standards were supplied by Romer (Tulin, Austria), except for zearalenone and its derivatives which were supplied by Sigma-Aldrich (Poznań, Poland)

#### 4.2.10. Yield Components 

Ripe seeds were collected, and the yield parameters were evaluated. Number and weight of seeds per spike were calculated in 45 replicates, the weight of a single seed was measured in 45 replicates, while the mass of one thousand seeds (MTS) was measured in three replicates, for each accession/treatment combination. 

### 4.3. Statistical Analyses

The experiments were arranged and performed with the application of a completely randomized design. The normal distribution of data was analyzed using Shapiro–Wilk test. Two-way analysis of variance (ANOVA) and Duncan’s multiple range test (at *p* < 0.05) were performed using the statistical package Statistica 13.3 (Stat–Soft, Inc., Tulsa, OK, USA). The data were presented as means ± SE (standard error). Pearson’s correlation coefficients were assumed as statistically significant at *p* < 0.05.

## 5. Conclusions

1. *Fusarium culmorum* infection significantly reduces the content of active photosynthetic pigments and the weight of leaves and roots.
2. The infected cv. ‘Tamaroi’ and BC_5_Nax_2_ plants recognized as more resistant to *F. culmorum* than SMH87, accumulated increased amounts of sugar in the leaves, which correlated with an increased number of phenolic compounds.
3. Phenolic compounds participate in H_2_O_2_ decomposition in durum wheat plants infected by *F. culmorum*.
4. The study confirmed the important role of H_2_O_2_ in increasing the content of phenolic compounds that are then incorporated into cell walls of plants infected with *F. culmorum.*
5. Nivalenol and deoxynivalenol secreted by *F. cumlorum* significantly reduce the yield of durum wheat.
6. Early evaluation of durum wheat spikes infection done seven days after inoculation with *F. culmorum* spores may help predict the potential degree of DON and NIV accumulation in the grain.

## Figures and Tables

**Table 1:** Pearson coefficients of linear correlation (*p* < 0.05) between the disease rating (DR) and chlorophyll *a, b* (Chl*a*, *b*), and carotenoid (Car) content and fresh weight (FW) of the leaves and roots.

Variable	Chl *a*	Chl *b*	Car	FW of Leaves	FW of Roots
DR in leaves	−0.859	−0.804	−0.870	−0.715	−0.755
DR in roots	−0.891	−0.811	−0.898	−0.672	−0.821

**Table 2:** Pearson coefficients of linear correlation (*p* < 0.05) between H_2_O_2_ content in the leaves and roots and the activity of catalase (CAT), peroxidases (POX), superoxide dismutase (SOD), as well as total soluble phenolic (TSP) and cell wall-bound phenolic (CWP) content in the leaves and roots of three accessions of durum wheat.

Variable	H_2_O_2_ Content	
	Leaves	Roots
CAT in leaves	0.710	ns
CAT in roots	ns	ns
POX in leaves	0.688	ns
POX in roots	ns	ns
SOD in leaves	ns	ns
SOD in roots	ns	0.613
TPC in leaves	−0.863	ns
TPC in roots	−0.763	−0.658
CWP in leaves	0.679	ns
CWP in roots	0.861	ns

ns—values not significant.

**Table 3:** *Fusarium* head blight index (FHBi) evaluated 7 and 14 days (DAI 7 and DAI 14) after spike inoculation with *F. culmorum* and yield components of three durum wheat genotypes.

Accession	Treatment	FHBi [%]		Amount of Grain Per Spike	Grain Mass Per Spike (g)	Mass of One piece of Grain (g)	MTS (g)
		DAI 7	DAI 14				
SMH87	Control	-	-	12.3 ± 1.4 ^b^	0.328 ± 0.048 ^b^	0.050 ± 0.003 ^b^	36.437 ± 0.319 ^b^
	Inoculum	10.1 ± 1.8 ^a^	34.7 ± 4.1 ^a^	2.0 ± 0.4 ^d^	0.055 ± 0.016 ^d^	0.012 ± 0.003 ^d^	15.603 ± 0.348 ^d^
Tamaroi	Control	-	-	16.8 ± 1.2 ^a^	0.459 ± 0.047 ^a^	0.049 ± 0.005 ^b^	37.674 ± 1.077 ^b^
	Inoculum	4.8 ± 0.9 ^b^	8.3 ± 1.1 ^b^	5.9 ± 1.0 ^d^	0.099 ± 0.019 ^d^	0.016 ± 0.004 ^d^	16.200 ± 1.853 ^d^
BC_5_Nax_2_	Control	-	-	13.6 ± 1.2 ^b^	0.355 ± 0.042 ^b^	0.061 ± 0.004 ^a^	51.763 ± 0.602 ^a^
	Inoculum	3.0 ± 0.7 ^b^	10.7 ± 1.9 ^b^	10.9 ± 1.1 ^c^	0.209 ± 0.023 ^c^	0.028 ± 0.004 ^c^	28.209 ± 1.905 ^c^

Values represent means ± SE. Fusarium head blight index (FHBi), amount of grain, grain mass per spike, and mass of one piece of grain were calculated in 45 replicates, while mass of one thousand seeds (MTS) were calculated in 3 replicates. Different superscript letters (a–d) FHBi and yield parameters indicate significant differences between means within columns (Duncan’s multiple range test; *p* < 0.05).

**Table 4:** Content [µg kg^−1^] of nivalenol (NIV), deoxynivalenol (DON^1^), T-2 toxin (T–2), and zearalenone (ZEN^2^) in the grain after *F. culmorum* spike inoculation.

Accession	Treatment	NIV	DON ^1^	ZEN ^2^	T-2
SMH87	Control	0.098 ± 0.008 ^d^	0.093 ± 0.002 ^c^	0.008 ± 0.001 ^e^	0.101 ± 0.003 ^e^
	Inoculum	1.785 ± 0.073 ^b^	5.763 ± 0.137 ^b^	0.002 ± 0.001 ^e^	0.166 ± 0.013 ^c^
Tamaroi	Control	0.414 ± 0.021 ^c^	0.037 ± 0.003 ^c^	0.004 ± 0.001 ^d^	0.136 ± 0.006 ^d^
	Inoculum	2.474 ± 0.109 ^a^	8.052 ± 0.373 ^a^	0.031 ± 0.001 ^b^	0.317 ± 0.010 ^a^
BC₅Nax₂	Control	0.406 ± 0.026 ^c^	0.047 ± 0.002 ^c^	0.022 ± 0.002 ^c^	0.126 ± 0.004 ^d^
	Inoculum	1.765 ± 0.018 ^b^	6.208 ± 0.318 ^b^	0.138 ± 0.002 ^a^	0.221 ± 0.005 ^b^

Values represent means (*n* = 3) ± SE. Different superscript letters (a–e) within columns indicate significant differences between means (Duncan’s multiple range test; *p* < 0.05). DON ^1^ total amount of deoxynivalenol (DON), and 3–acetyldeoxynivalenol (3AcDON) and 15–acetyldeoxynivalenol (15AcDON); ZEN ^2^ total amount of zearalenone (ZEN); alpha–Zearalanol (α–ZAL), beta–Zearalanol (β–ZAL); alpha–Zearalanol (α–ZEL) and beta–Zearalenol (β–ZEL).

**Table 5:** Pearson’s coefficients of linear correlation (*p* < 0.05) between *Fusarium* head blight index (FHBi) evaluated 7 and 14 days after inoculation (DAI 7 and DAI 14), mycotoxin content in grain and yield parameters.

Variable	FHBi DAI 7	FHBi DAI 14	NIV	DON ^1^	ZEN ^2^	T–2
Number of grain per spike	−0.591	−0.575	−0.503	−0.549	ns	ns
Mass of grain per spike [g]	−0.589	−0.576	−0.566	−0.612	ns	ns
Mass of a single grain [g]	−0.754	−0.743	−0.863	−0.864	−0.714	ns
MTS [g]	−0.741	−0.848	−0.551	−0.589	ns	ns

DON ^1^ total amount of deoxynivalenol (DON), 3–acetyldeoxynivalenol (3AcDON) and 15–acetyldeoxynivalenol (15AcDON); ZEN ^2^ total amount of zearalenone (ZEN); alpha–Zearalanol (α–ZAL), beta–Zearalanol (β–ZAL); alpha–Zearalanol (α–ZEL) and beta–Zearalenol (β–ZEL); MTS-mass of thousand seeds; ns—values not significant.

**Table 6:** Pearson’s coefficients of linear correlation (*p* < 0.05) between *Fusarium* head blight index (FHBi) evaluated 7 and 14 days after the inoculation (DAI 7 and DAI 14) and mycotoxin content in the grain.

Variable	NIV	DON ^1^	ZEN ^2^	T-2 Toxin
FHBi DAI 7	0.731	0.733	0.484	ns
FHBi DAI 14	0.630	0.632	ns	ns

DON ^1^ total amount of deoxynivalenol (DON), 3–acetyldeoxynivalenol (3AcDON) and 15–acetyldeoxynivalenol (15AcDON); ZEN ^2^ total amount of zearalenone (ZEN); alpha–Zearalanol (α–ZAL), beta–Zearalanol (β–ZAL); alpha–Zearalanol (α–ZEL) and beta–Zearalenol (β–ZEL); MTS-mass of thousand seeds; ns—values not significant.

## Data Availability

All data relevant to the main findings of this study are included within the article and supplementary material.

## Supplementary Materials

The following are available online at https://www.mdpi.com/article/10.3390/ijms22147433/s1, Table S1: Multiple reactions monitoring (MRM) transitions for the analyzed mycotoxins at positive ion mode (+ESI), capillary voltage 4 kV, gas temperature 300 °C, gas flow 12 L/min and nebulizer pressure 35 psi. MassHunter software was used to control the UHPLC–MS/MS system and in data analysis. For MRM parameters optimization MassHunter Optimizer was used.
