# Supplementary material for: Physiological and Biochemical Response to Fusarium culmorum Infection in Three Durum Wheat Genotypes at Seedling and Full Anthesis Stage
Source: Int J Mol Sci. 2021 Jul 11;22(14):7433. doi: 10.3390/ijms22147433 (PMC8303160; doi:10.3390/ijms22147433)
Supplement: Supplementary file 1 [file ijms-22-07433-s001.zip › ijms-1285082-supplementary.pdf]

Table S1. Multiple reactions monitoring (MRM) transitions for the analyzed mycotoxins at positive ion mode (+ESI), capillary voltage 4 kV, gas temperature 300 °C, gas flow 12 l/min and nebulizer pressure 35 psi. MassHunter software was used to control the UHPLC–MS/MS system and in data analysis. For MRM parameters optimization MassHunter Optimizer was used.

		Type of ion	Precursor ion	Quantifier product ion	Qualifier product Ion	Fragmentor voltage (V)	Collision energy (V)	Retention Time (min)
Nivalenol	NIV	$[M+H]^+$	295.2	137	91.1	102	13; 49	1.05
Deoxynivalenol	DON	$[M+H]^+$	297.2	249.1	77.1	79	9; 69	1.8
3-acetyldeoxynivalenol and 15-acetyldeoxynivalenol	3+15AcDON	$[M+H]^+$	339.2	261.1	203.1	84	9; 13	3.2
Alpha-zearalanol	aZAL	$[M+H]^+$	305.2	189.1	123	145	13; 29	5.1
Beta- zearalanol	bZAL	$[M+H]^+$	305.2	189.1	123	145	13; 29	4.6
Alpha zearalenol	aZEL	$[M+H]^+$	303.2	285.1	91.1	114	9; 61	5.3
Beta-zearalenol	bZEL	$[M+H]^+$	303.2	285.1	91.1	114	9; 61	4.8
T2- toxin	T2	$[M+H]^+$	484.2	215.2	185.1	114	13; 21	5.4
Zearalenone	ZEN	$[M+H]^+$	319.2	301.1	283.1	107	9; 9	5.75
